# Invasive Mechanical Ventilation in California Over 2000–2009: Implications for Emergency Medicine

**DOI:** 10.5811/westjem.2015.6.25736

**Published:** 2015-10-20

**Authors:** Seshadri C. Mudumbai, Juli Barr, Jennifer Scott, Edward R. Mariano, Edward Bertaccini, Hieu Nguyen, Stavros G. Memtsoudis, Brian Cason, Ciaran S. Phibbs, Todd Wagner

**Affiliations:** * Anesthesiology and Perioperative Care Service, Veterans Affairs Palo Alto Health Care System; † Stanford University School of Medicine, Department of Anesthesiology, Perioperative and Pain Medicine, Stanford, California; ‡ Center for Innovation to Implementation, Veterans Affairs Palo Alto Health Care System; § George Washington School of Medicine, Washington, DC; ¶ Hospital for Special Surgery, Weill Medical College of Cornell University; || Anesthesia Service, Veterans Affairs San Francisco Health Care System; # University of California, San Francisco, Department of Anesthesiology and Perioperative Care, California; ** Stanford University School of Medicine, Department of Pediatrics

## Abstract

**Introduction:**

Patients who require invasive mechanical ventilation (IMV) often represent a sequence of care between the emergency department (ED) and intensive care unit (ICU). Despite being the most populous state, little information exists to define patterns of IMV use within the state of California.

**Methods:**

We examined data from the masked Patient Discharge Database of California’s Office of Statewide Health Planning and Development from 2000–2009. Adult patients who received IMV during their stay were identified using the International Classification of Diseases 9th Revision and Clinical Modification procedure codes (96.70, 96.71, 96.72). Patients were divided into age strata (18–34yr, 35–64yr, and >65yr). Using descriptive statistics and regression analyses, for IMV discharges during the study period, we quantified the number of ED vs. non-ED based admissions; changes in patient characteristics and clinical outcome; evaluated the marginal costs for IMV; determined predictors for prolonged acute mechanical ventilation (PAMV, i.e. IMV>96hr); and projected the number of IMV discharges and ED-based admissions by year 2020.

**Results:**

There were 696,634 IMV discharges available for analysis. From 2000–2009, IMV discharges increased by 2.8%/year: n=60,933 (293/100,000 persons) in 2000 to n=79,868 (328/100,000 persons) in 2009. While ED-based admissions grew by 3.8%/year, non-ED-based admissions remained stable (0%). During 2000–2009, fastest growth was noted for 1) the 35–64 year age strata; 2) Hispanics; 3) patients with non-Medicare public insurance; and 4) patients requiring PAMV. Average total patient cost-adjusted charges per hospital discharge increased by 29% from 2000 (from $42,528 to $60,215 in 2014 dollars) along with increases in the number of patients discharged to home and skilled nursing facilities. Higher marginal costs were noted for younger patients (ages 18–34yr), non-whites, and publicly insured patients. Some of the strongest predictors for PAMV were age 35–64 years (OR=1.12; 95% CI [1.09–1.14], p<0.05); non-Whites; and non-Medicare public insurance. Our models suggest that by 2020, IMV discharges will grow to n=153,153 (377 IMV discharges/100,000 persons) with 99,095 admitted through the ED.

**Conclusion:**

Based on sustained growth over the past decade, by the year 2020, we project a further increase to 153,153 IMV discharges with 99,095 admitted through the ED. Given limited ICU bed capacities, ongoing increases in the number and type of IMV patients have the potential to adversely affect California EDs that often admit patients to ICUs.

## INTRODUCTION

Management of patients requiring invasive mechanical ventilation (IMV) often represents a sequence of care starting with the pre-hospital period, extending to emergency department (ED) management and peaking with intensive care unit (ICU) admission and treatment i.e., the “critical care cascade.” 1 For patients who require IMV, initial presentation to ED may involve medical stabilization with a trial of non-IMV. 2 The patient who fails initial management may then require emergent intubation, mechanical ventilation, and eventual transfer to the ICU. 2 Studies have already documented an increase in ICU admissions from the ED over the past decade on a national level.^3^,^4^

For the estimated 800,000 adult patients who require IMV annually in the United States (U.S.), acute respiratory failure remains one of the most common indications. 5 Increasing age, the presence of co-morbidities (i.e., chronic obstructive pulmonary disease, congestive heart failure, asthma), and acuity of illness are all independent predictors of the need for IMV in patients with acute respiratory failure.^6^,^7^ Although IMV can be a life-saving intervention, it is associated with major costs estimated at $27 billion annually in the U.S. alone. 5 Aggravating this problem is the fact that while patients who require prolonged acute mechanical ventilation (PAMV; defined as IMV≥96 hr) make up less than 10% of the adult IMV patient population, they can account for two-thirds of all annual hospital costs associated with IMV. 5, 8 The incidence, duration, and costs associated with IMV in the U.S. are only expected to increase substantially over the next several decades as the U.S. population ages and the co-morbidity burden of patients with acute respiratory failure rises.^5^,^9^–^11^

Despite being the most populous state (38 million) in the nation little information exists to define patterns of IMV use within the state of California.^12^,^13^ California employs substantial data documenting capabilities allowing for analysis of state-level variation in healthcare. Furthermore, with data already indicating an increasing utilization of critical care services within California EDs for the past decade, population-based analyses of IMV usage within the state may be useful to identify future policy priorities. 14 The objectives of this study were to 1) identify the number of ED vs. non-ED based admissions, demographic patterns, outcomes, and marginal costs of patients who underwent IMV; 2) determine risk factors for prolonged IMV; and 3) predict future IMV usage in the state of California.

## METHODS

### Study Design And Principal Data Source

Given this study is an analysis of publicly available data and de-identified data are used, the study was deemed exempt by our university-affiliated institutional review board. We examined retrospective data from the masked Patient Discharge Database (PDD) obtained from California’s Office of Statewide Health Planning and Development (OSHPD) for the period 2000–2009. For the past three decades, OSHPD has mandated that all California hospitals collect and report detailed information on all patients whose hospital stay is >1 day. The masked PDD contains information on all patient discharges from non-federal, acute-care hospitals, with standardized, random masking of key demographic variables to prevent linkages of patients across discharges. For example, across the age strata used within our sample, OSHPD masks data at the same frequency, i.e. 6–8%.

For this analysis, we used PDD variables from the following categories: OSHPD-hospital identification number; the county and zip code of each hospital; year of admission; patients’ age in years upon admission; admission source; gender; race/ethnicity; expected principal source of payment (i.e., plan code number); principal procedure code fields (including the principal procedure, and up to 24 additional procedures); hospital charges and discharge disposition (i.e., for both medical and surgical patients to home, skilled nursing facility, etc). OSHPD closely monitors the quality of its data reporting with low levels (<0.1%) of missing data for the variables used for this study. In addition, data extracts are released yearly only after screening by automated reporting software and correction by individual facilities. OSHPD’s data standardization has enabled population level and hospital quality of care analyses such as system-level health disparities in California EDs.^15^,^16^

### Study Population

**Figure 1:**
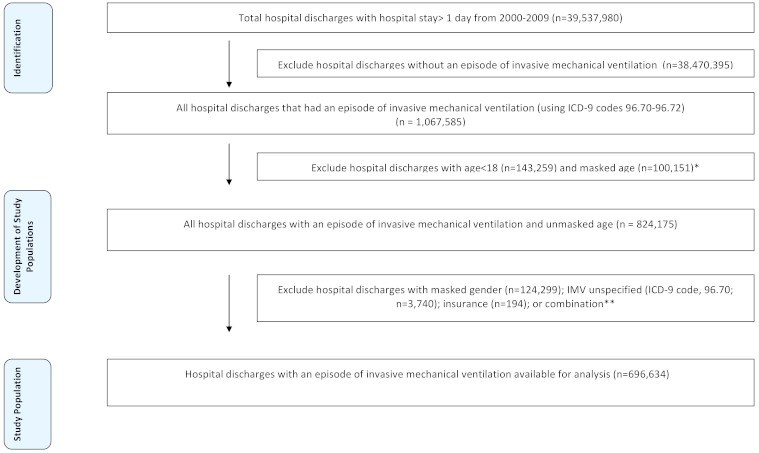
Selection of study population. *IMV,* invasive mechanical ventilation; *ICD-9,* International Classification of Diseases 9^th^ Revision *Number of IMV unspecified discharges (n=569). **Some patients with IMV unspecified (ICD-9 code:96.70) also had missing gender or some combination of those three variables (IMV time, gender, and insurance).

After identifying discharges with hospital stay >1 day from 2000–2009, our initial sample consisted of 39,537,980 patient discharges (Figure 1). Our objective was to identify only those patients who underwent IMV at any point during their hospital stay for the study period. To accomplish this, we initially screened all patients with the International Classification of Diseases 9^th^ Revision and Clinical Modification (ICD-9-CM) procedure codes 96.70 (mechanical ventilation, unspecified), 96.71 (mechanical ventilation for <96 hours), or 96.72 (mechanical ventilation for ≥96 hours) in their discharge records, resulting in an initial sample of 1,067,585 discharges. Professional coders, not physicians, create the ICD-9 codes; audits have shown these data to be very accurate. 17 We then excluded records with age<18, masked age, gender, insurance type, or unspecified duration of mechanical ventilation (n=3,740), leaving us with a final sample of n=696,634 discharges for analysis.

### Data Collection

Patients were initially divided into three broad age strata based upon their age at the time of admission: 18–34yr, 35–64yr, and ≥65yr. We identified the number of IMV patients who were admitted through the ED. Patients were classified by gender (male vs. female), and race/ethnicity (White, Hispanic, Black, Asian, other, or unknown). Insurance was categorized as public (Medicare vs. non-Medicare public: Medi-Cal [California’s Medicaid program], county indigent programs), private, or other (worker’s compensation, self-pay, and other payer). To account for differences in the distribution of patients’ co-morbidities, we constructed a Charlson illness severity index (using the Charlson-Deyo-Quan method) for each patient using all discharge diagnosis codes. 18 We aggregated patients’ illness severity scores into levels (0 to 3+) on the basis of sample distribution; higher scores represented a greater severity of illness. To identify surgical admissions, principal procedure codes for each discharge were merged with Healthcare Cost and Utilization Project (HCUP) identifiers to distinguish surgical procedures. 19 HCUP classifies ICD-9 procedure codes as minor diagnostic, minor therapeutic, major diagnostic, and major therapeutic; major diagnostic and therapeutic codes refer to procedures routinely conducted in the OR. Major diagnostic and therapeutic ICD-9 codes were selected to identify operating room surgical procedures. For the set of patients who were not admitted through the ED, we quantified the number of surgical vs. medical patients.

Hospitals were classified by urban vs. rural geographic location using rural-urban commuting area (RUCA) code linked to each hospital’s zip code. The RUCA codes use data from U.S. census tracts on measures of population density, urbanization, and daily commuting to classify zip codes. We used the most recent RUCA codes (2006 ZIP Version 2.0 last updated 11/13/07), based on 2000 US Census data. We used the following codes: Urban: 1.0, 1.1, 2.0, 2.1, 3.0, 4.1, 5.1, 7.1, 8.1, 10.1; Rural: 4.0, 4.2, 5.0, 5.2, 6.0, 6.1, 7.0, 7.2, 7.3, 7.4, 8.0, 8.2, 8.3, 8.4, 9.0, 9.1, 9.2, 10.0, 10.2, 10.3, 10.4, 10.5, 10.6.

### Outcomes

Outcomes of interest included death, discharge status (home, acute care hospital, other care hospital, or skilled nursing facility), lengths of stay and total hospital costs. We estimated total costs by adjusting hospital charges using available cost-to-charge ratios from Centers for Medicaid and Medicare Impact files. All costs were also adjusted to 2014 dollars using the Consumer Price Index and to reflect stays more than one year in length.

### Statistical Analyses

For years 2000–2009, we used data collected by the California Department of Finance to calculate a California population growth rate relative to all hospitalizations in the population from 2009. This value was then applied to counts of discharges in year 2000.

We aggregated patient and hospital characteristics, and clinical outcomes using descriptive statistics and t-tests, analysis of variance, or chi square tests as appropriate. Initial comparisons for patient characteristics were also based on unadjusted logistic regressions. First, we conducted an ordinary least squares linear regression analysis with robust standard errors to evaluate marginal costs for IMV. Marginal costs for IMV are defined as the average incremental cost of mechanical ventilation per discharge. All independent factors were used for model development and were forced into the model: age strata, gender, race/ethnicity, insurance, co-morbidity burden, surgery, and hospital geographic location. Second, we conducted a multivariate logistic regression analysis that predicted PAMV (i.e. IMV≥96 hr) using the same independent factors included in our marginal cost model. Third, based on average growth rates of IMV from 2000–2009, we projected rates of IMV discharges and ED-based admissions for the year 2020 using linear regression. A p-value≤0.05 was considered statistically significant (two-sided tests). We used SAS software, version 9.2 (SAS Institute Inc., Cary, NC) for the statistical analyses and Stata 12.1 (Stata Corporation, College Station, TX, USA) for figures.

## RESULTS

### Demographic Patterns And Outcomes Of IMV

**Figure 2:**
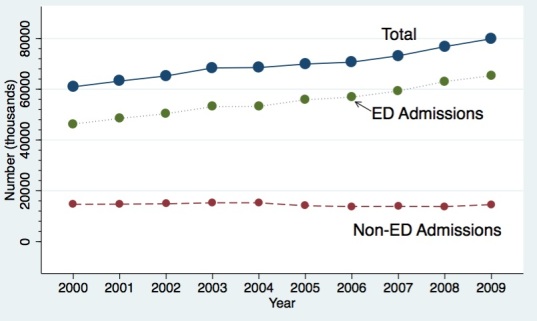
Invasive mechanical ventilation (IMV) discharges in California from 2000–2009. *ED*, emergency department

From 2000–2009 (n=696,634 IMV discharges), we noted an absolute increase from n=60,933 IMV discharges (293 IMV discharges/100,000 persons) in year 2000 to n=79,868 (328 IMV discharges/100,000 persons) in year 2009 (average yearly growth rate=+2.8%) (Figure 2). IMV discharges originating from the ED also increased in parallel fashion from n=46,258 in 2000 to n=65,321 in 2009: a 3.8% annual growth rate (Figure 2). Non-ED admissions had a 0% growth rate (n=14,675 in 2000 to n=14,547 in 2009). For ED-based admissions during the study interval, the largest increase was noted in medical patients (from n=32,722 to 46,173), not surgical patients (from 13,516 to 19,144).

Table 1 summarizes the characteristics of the overall study population, and for years 2000 and 2009. The following growth rates are reported as average yearly rates and are absolute (not population adjusted). Overall, our study population primarily was older (≥65 year strata; 54.9%); almost equally divided between female and male; primarily White (58.3%); receiving Medicare insurance (55.4%); over one third of patients had three or more co-morbidities; with a medical admission (65.1%); urban (95.5%); and with a mechanical ventilation time <96 hours. During 2000–2009, fastest growth was noted for 1) the 35–64 year age strata (+4.7%/year) vs. the ≥65 year strata (+1.2%/year); p<0.01; 2) Hispanics:(+6.8%/year) vs. Whites (+ 1.0%/year); p<0.01; and 3) non-Medicare patients with public insurance vs. Medicare patients (+2.5%/year); p<0.01. The proportion of patients requiring PAMV (i.e. IMV>96hr) also increased fastest over time (+3.8%/year), with increases noted for all age strata vs. IMV<96hr (+ 2.3%/year); p<0.01.

Clinical outcomes and total hospital costs are shown in Table 2. For the entire study population, approximately one third died in hospital. We noted increases upon hospital discharge in the number of patients who were discharged to home (2000:13/1,000 IMV discharges to 2009:17/1,000 IMV discharges) and transferred to skilled nursing facilities (2000:10/1,000 IMV discharges to 2009:16/1,000 IMV discharges). While the average hospital length of stay (LOS) was 14d for all patients receiving IMV, hospital LOS increased over the study period, especially for survivors (0.6d). Decedents had an average hospital LOS of 11.2d, which did not decrease over time. From 2000–2009, average total patient cost-adjusted charges per hospital discharge with an IMV episode increased by 29% from 2000 (from $42,528 to $60,215 in 2014 dollars) with increases noted for both survivors and decedents. PAMV patients had an approximately three-fold difference in average costs overall and for 2000 and 2009. Table 3 shows that higher marginal costs for IMV were noted for patients who were younger (both age 18–34 years and 35–64 strata), male and non-White, had non-Medicare public insurance, had a higher co-morbidity burden, a surgical admission, and those hospitalized in urban hospitals. Marginal costs increased progressively each year with an approximate four-fold difference between 2000 and 2009 ($3,590 to $16,898 in 2014 dollars; p<0.05).

### Risk Factors For Prolonged Acute Mechanical Ventilation

Factors associated with an increased probability of PAMV are provided in Table 4. The strongest predictors for PAMV were: age 35–64yr (OR=1.12; 95% CI [1.09–1.14], p<0.01); non-Whites: Hispanic (OR=1.08; 95% CI [1.07–1.10], p<0.01), Black (OR=1.12; 95% CI [1.10–1.14], p<0.01) and Asian (OR=1.22; 95% CI [1.19–1.24], p<0.01); non-Medicare public insurance (OR=1.18; 95% CI [1.18–1.16–1.20]; p<0.01); increasing co-morbidity burden; surgical admission; an urban hospitalization, and by the end of the study period.

### Projected IMV Use

By 2020, our models suggest that IMV utilization would increase to 153,153 IMV discharges (377 IMV discharges/100,000 persons) of which 99,095 would be admitted through the ED.

## DISCUSSION

In this population level study of IMV use in California, based on sustained growth over the past decade, by the year 2020, we project a further increase to 153,153 IMV discharges (377 IMV discharges/100,000 persons) with 99,095 admitted through the ED. During the study interval, fastest IMV growth rates were observed in patients who were admitted through the ED, and in middle-aged and Hispanic cohorts. While more IMV patients were discharged to home and skilled nursing facilities, there were tendencies towards prolonged mechanical ventilation and longer hospitalizations. Our study is one of the first to document patterns of IMV usage in California while using a longitudinal approach with cost analyses. Since patients who require IMV often represent a sequence of care between the ED and ICU, if changes in the volume and type of IMV patients are sustained for the near future, these factors, along with a limited ICU bed capacity, have the potential to create substantial, additional strains on California EDs.^1^,^2^,^4^,^14^

### Demographic Patterns of IMV

The dramatic increase in the overall number of IMV-discharges and related costs in California over the past decade may be attributed to multiple factors, including California’s population growth, increasing use of critical care resources, and advances in the management of coexisting conditions during this period.^5^,^9^,^10^ From 2000–2009, California’s population grew by 10% from roughly 34 million to 37 million. 20 Demographic projections also suggest that older patients (≥65yr) will increase from 10% of California’s population in 2000 to 20% by 2020, a growth rate similar to the U.S. overall. 21 In response to both population growth and healthcare financing changes, hospitals throughout California implemented cost-cutting strategies by moving procedures to outpatient settings and the creation of more ICU beds (with numbers stabilizing in the late 2000s).^22^,^23^ The net effect has been to have sicker patients in hospitals with more consumption of ICU beds. We noted an overall increase in the percentage of patients with the highest co-morbidity burden (i.e. Charlson index of 3) over the study period.

Simultaneously, average total costs for IMV patients in California increased by 42% over the 10-year study period, more than double the growth rate of California’s gross domestic product (GDP) (19%) and the U.S. GDP (16%) during this same period. 24 Increasing costs for an IMV-discharge were noted for the overall study population, across all age groups, and for both survivors and decedents. While potential reasons for this growth in costs include worsening co-morbidity burdens for critically ill patients, other reasons might be advances in critical care medicine promoting survivorship (i.e. automatic weaning strategies) and the less prominent role of palliative care at the time.^25^,^26^

Although older patients (≥65 years) still accounted for the majority of patients overall receiving IMV in CA from 2000–2009, IMV use grew fastest during the same time period for younger age groups, particularly those age 35–64 years (from 19 to 32 discharges/1,000 or a growth rate of +4.7%/year). In our multivariate analysis of marginal costs, our models also showed highest marginal costs for IMV among those aged 35–64 years and 18–34 years. Our regional findings differ from national level data predicting that greatest growth rates in mechanical ventilation will occur in older patients as the “baby boomer” generation begins to pass age 65 in the U.S. 5 We speculate that the observed age-related patterns in IMV usage in this study may represent a shift towards less aggressive treatments for older patients at the end of life, together with a growing acuity of younger patients due to an increasing co-morbidity burden and a potential lack of access to healthcare in California.^27^,^28^

IMV use also increased faster for all non-White ethnic minorities, especially Hispanics and Blacks. Increased marginal costs for an IMV episode were indeed found for Hispanic and Black patients. One potential explanation may be the dramatic increase in population growth for all ethnic minorities in California. Hispanics represent the fastest growing segment of the state’s population due in large part to immigration; in addition, immigrant populations in California tend to be ethnic minorities, younger, clustered in urban areas, and lack private insurance.^15^,^28^ The growth of IMV in ethnic minorities is consistent with our data documenting an increase in IMV in younger age strata, in urban hospitals, and those with non-Medicare public (i.e. Medi-Cal) insurance.

### Growth and Risk Factors For PAMV

The observed regional level growth rates for PAMV in our study are similar to previously predicted national growth rates for PAMV.^8^,^29^ Our models showed an increased odds of PAMV for the end of the study period relative to the start (2009: OR: 1.14, 95% CI=1.09–1.14, p<0.01). Increasing rates of PAMV likely reflect both improvements in critical care as well as an increase in the co-morbidity burden of patients over time. 30 We found that IMV patients with three or more co-morbidities were nearly twice as likely as those with a Charlson index of zero to require prolonged mechanical ventilation. In addition, data indicate that critically ill patients who have received a prolonged course of IMV are more likely to suffer additional iatrogenic complications; have longer hospital lengths of stay; have higher in-hospital and one-year post-discharge mortality rates; and a higher incidence of long-term physical and cognitive disability, leading to a higher proportion of these patients being discharged from the hospital to a skilled nursing facility. 31 We noted an increase in the hospital LOS for all IMV patients and the proportion of patients discharged to skilled nursing facilities.

The age strata 35–64 years had higher odds of prolonged mechanical ventilation in this study than older IMV patients. Potential explanations include a trend towards earlier transitions to comfort care measures and an earlier withdrawal of life support in high acuity elderly patients over time. 32 In addition, all ethnic minorities had higher odds of prolonged mechanical ventilation compared with Whites. Based on the similarity in results on overall IMV usage (i.e., increase in IMV over the study period for ethnic minorities, urban hospitals, and those lacking private insurance), further research is necessary to identify whether factors such as co-morbidity burden or healthcare access may be also contributing to an increased risk of PAMV for these vulnerable populations. 33

### Policy Implications

If trends continue for an increasing number of IMV episodes, for rising admissions through EDs, and for growing IMV utilization by younger patients, ethnic minorities, and those requiring PAMV, then all of these factors will place an enormous stress on California EDs. 34 Asplin’s widely used input-throughput-output conceptual model for ED patient flow can be helpful in identifying where these trends may most affect California EDs.^35^ Briefly, this model suggests that EDs operate within the context of a greater hospital milieu with input describing elements such as safety-net care affecting demand for ED care; throughput defining operations within the ED such as boarding of inpatients; and output identifying variables such as inpatient bed occupancy rates affecting transfer and discharge of patients. Based on Asplin’s model, we suggest two immediate impacts by IMV patients on ED care processes.

First, an increased number of IMV episodes and a relatively fixed ICU bed capacity in California may create higher ICU bed occupancy rates. In turn, higher ICU bed occupancy rates along with a growing number of ED-based admissions for IMV patients can affect ED output and increase ED boarding times. Because of potential hospital-mandated nurse-patient 1:1 staffing ratios for IMV patients, crucial nursing resources may be unavailable to process and manage less acute patients further aggravating ER crowding and waiting times.^14^ As IMV patients experience increased ER boarding times, emergency physicians (EP) may also be called upon to manage a larger proportion of mechanically ventilated patients for longer intervals.^22^,^36^,^37^ However, data indicate that EPs may be less comfortable managing ventilator settings and monitoring progression to acute lung injury for IMV patients.^36^ Extended LOS for IMV patients in ED settings have also been associated with poorer outcomes.^2^,^37^

Second, with ongoing growth in IMV episodes by younger patients, ethnic minorities, and those with non-Medicare public insurance, Asplin’s model suggests additional, large increases in demand for care in EDs that serve these patient populations. Data indicate that California EDs as a whole already serve a large proportion of minority and Medicaid populations for safety net care.^15^ According to the Agency for Healthcare Research and Quality, ethnic minorities are more likely to be near or below the poverty line than Whites, are less likely to have health insurance, and are 20–60% more likely to experience significant barriers in their access to quality healthcare.^33^ As a result, ethnic minorities tend to have a greater co-morbidity burden and experience significant delays in receiving timely, high quality healthcare, resulting in a higher average acuity for minority patients at the time of hospital and ED admission.^15^ Our data showed that the largest increase in ED-based admissions was in medical not surgical patients. ED crowding may be intensified by the influx of patient presenting with acute respiratory failure; reports already indicate that high ED crowding is associated with increased inpatient mortality.^38^

## LIMITATIONS

Our study has several potential limitations. First, we used administrative data to examine patient discharges with IMV usage, and coding errors could have occurred. We also lacked clinical details on patient management with consequent inability to look at complications and events after discharge. However, both IMV and PAMV coding have been noted to have very good inter-rater reliability.^18^ In addition, our goal was to generate a descriptive analysis of patterns for IMV and to inform policy discussions. Second, our analysis was restricted to California, potentially limiting generalizability to other parts of the U.S. or other countries. Nevertheless, we examined decade-long patterns from the most populated state, and compared discharges for many hospitals over different age strata. While our results may not be immediately generalizable to the U.S. as a whole, our methods give a framework for other states to use when looking at their states data and future needs. Third, future studies are necessary to better estimate marginal costs for IMV using more homogeneous subgroups of patients (i.e., by disease) and better account for post-discharge care for these patients. Finally, our study used the masked PDD, so we were unable to account for correlations for repeat admissions for the same patient. We excluded about 224,000 patients (25% of our initial sample) for masked age and masked gender. However, we were able to use a substantially large sample from a systematically de-identified dataset. The large OSHPD database also provides the ability to perform a population-level analysis that includes patients in multiple types of ED and hospital settings (e.g. tertiary, academic, community settings).

## CONCLUSION

Based on sustained growth over the past decade, by the year 2020, we project a further increase to 153,153 IMV discharges with 99,095 admitted through the ED. Given our projections for a steady, substantial growth of IMV discharges within California over the next five years along with potential ED-based admissions, our main findings suggest the need for healthcare management strategies that target younger patients, ethnic minorities, and patients requiring prolonged mechanical ventilation. While longer-term goals include improved outcomes for these vulnerable patients and reducing healthcare-related expenditures, short-term policy priorities would involve modeling the impact of increased number of IMV patients on California EDs.^8^,^39^ More research is needed to confirm our main findings with additional lines of research to determine necessary levels of ED staffing, strategies to decrease ER boarding times, and to quantify resource allocation for safety net EDs.

## Figures and Tables

**Table 1:** Demographic and clinical characteristics of invasive mechanical ventilation (IMV) discharges in California, 2000–2009.

Characteristic	All patients from 2000–2009 (n=696,634)#	2000*	2009*
Age strata			
18–34 (yrs)	47,371 (6.8)	4	6
35–64 (yrs)	266,114 (38.2)	19	32
≥65 (yrs)	382,452 (54.9)	33	42
Gender,			
Female, reference	328,115 (47.1)	27	37
Male	368,519 (52.9)	29	43^a^
Race/ethnicity			
White, reference	406,138 (58.3)	36	43
Hispanic	100,315 (14.4)	7	13^a^
Black	57,124 (8.2)	4	7^a^
Asian	39,012 (5.6)	3	5^a^
Other	11,843 (1.7)	1	2^a^
Unknown	82,203 (11.8)	6	10^a^
Type of insurance			
Medicare, reference	385,935 (55.4)	32	44
Non-Medicare public	138,630 (19.9)	10	17^a^
Private	133,057 (19.1)	12	15^a^
Other	39,012 (5.6)	3	5^a^
Charlson co-morbidity index			
0, reference	101,709 (14.6)	8	11
1–2	345,530 (49.6)	30	38 a
3+	249,395 (35.8)	17	31 a
Surgery			
No, reference	453,509 (65.1)	36	53
Yes	243,125 (34.9)	20	27^a^
Urban vs. rural			
Urban, reference	665,285 (95.5)	53	77
Rural	20,899 (3)	2	2
Unknown	10,450 (1.5)	1	1
Mechanical ventilation time			
<96 hours, reference	413,801 (59.4)	35	47^a^
≥96 hours	282,833 (40.6)	21	33

# Displayed as count (column percent).

* Displayed as number of patients per 1,000 IMV discharges. All counts for the year 2000 are population adjusted relative to all 2009 hospitalizations in the California population.

a P-values<0.01 based on logistic regression models comparing 2009 discharges with 2000, for all discharges and within each age group.

**Table 2:** Outcomes of patients who received mechanical ventilation in California, 2000–2009.

Characteristic	2000–2009	2000	2009
Discharge status			
Home	151,866 (21.6)#	13*	17*
Acute care hospital	54,337 (7.8)#	5*	6*
Other care hospital	39,708 (5.7)#	3*	5*
Skilled nursing facilities	130,271(18.7)#	10*	16*
In-hospital death	246,068 (35.4)#	20*	27*
Other	67,573 (9.7)#	5*	9*
Unknown	6,270 (0.9)#	0*	1*
Days of stay, mean			
All patients	14.1	13.4	13.9
Survivors	15.7	14.8	15.4
Decedents	11.2	10.9	10.8
Total cost** average cost per patient			
All patients	54,931	42,528	60,215
Survivors	58,566	44,800	64,122
Decedents	48,311	38,595	52,482
Total cost** average cost per patient by mechanical ventilation time			
<96 hours	27,708	21,575	31,002
≥96 hours	82,105	66,018	88,001

# Displayed as absolute counts (column percent)

* Displayed as number of patients per 1,000 IMV discharges. All counts for the year 2000 are population adjusted relative to all 2009 hospitalizations.

** Total costs were estimated by adjusting hospital charges using available cost-to-charge ratios. All costs were also adjusted to 2014 dollars using the Consumer Price Index and to reflect stays more than one year in length.

**Table 3:** Marginal cost per invasive mechanical ventilation discharge in California, 2000–2009.

Characteristic	Marginal cost
Age group†	
18–34	Reference
35–64	−1,280‡
≥65	−8,820‡
Gender†	
Female	Reference
Male	2,743‡
Race/ethnicity†	
White	Reference
Hispanic	417
Black	10,560‡
Asian	3,104‡
Other	−118
Unknown	8,026‡
Insurance†	
Medicare	Reference
Other public	17,248‡
Private	−5,355‡
Other	−5,913‡
Charlson comorbidity index†	
0	Reference
1–2	8,349‡
≥3	14,537‡
Surgery†	
No	Reference
Yes	60,443‡
Urban/rural†	
Urban	Reference
Rural	−13,662‡
Unknown	10,536‡
Discharge year†	
2000	Reference
2001	3,590‡
2002	8,694‡
2003	11,904‡
2004	13,936‡
2005	13,085‡
2006	14,983‡
2007	14,668‡
2008	16,898‡
2009	

Intercept=$12,885.

* Total costs were estimated by adjusting hospital charges with available cost-to-charge ratios. All costs were adjusted to 2014 dollars; we used the Consumer Price Index to reflect stays that spanned more than one year.

† P-value significant at 0.05 for overall F-test.

‡ P-value significant at 0.05 for contrast with reference group from multivariate linear regression model with robust standard errors.

**Table 4:** Risk factors for prolonged acute mechanical ventilation in California, 2000–2009.

Characteristic	Odds ratio (95% CI)
Age group	
18–34	Reference
35–64	1.12 (1.09, 1.14)†
≥65 years	1.03 (1.00, 1.05)‡
Gender	
Female	Reference
Male	1.05 (1.04, 1.06)†
Race/ethnicity	
White	Reference
Hispanic	1.08 (1.07, 1.10)†
Black	1.12 (1.10, 1.14)†
Asian	1.22 (1.19, 1.24)†
Other	1.10 (1.06, 1.14)†
Unknown	1.13 (1.11, 1.15)†
Type of insurance	
Medicare	Reference
Other public	1.18 (1.16, 1.20)†
Private	0.87 (0.86, 0.88)†
Other	0.71 (0.70, 0.73)†
Charlson comorbidity index	
0	Reference
1–2	1.58 (1.55, 1.60)†
3+	1.99 (1.96, 2.03)†
Surgery	
No	Reference
Yes	2.08 (2.06, 2.10)†
Urban/rural	
Urban	Reference
Rural	0.62 (0.60, 0.64)†
Unknown	0.85 (0.82, 0.89)†
Discharge year	
2000	Reference
2001	1.03 (1.00, 1.05)‡
2002	1.05 (1.02, 1.07)†
2003	1.07 (1.05, 1.10)†
2004	1.09 (1.06, 1.11)†
2005	1.11 (1.08, 1.13)†
2006	1.12 (1.10, 1.15)†
2007	1.14 (1.11, 1.16)†
2008	1.14 (1.11, 1.16)†
2009	1.11 (1.09, 1.14)†

Prolonged acute mechanical ventilation (PAMV): invasive mechanical ventilation (IMV) ≥96 hours (n=282,664); Reference group is IMV<96 hours (n=413,970)].

† P-value<0.01.

‡ P-values<0.05.
